# Genetic disorders and congenital anomalies in Nigeria: a scoping review

**DOI:** 10.1007/s12687-026-00876-w

**Published:** 2026-04-09

**Authors:** Ayoade Desmond Babalola, Nitza Ferreira Muniz, Lavinia Schuler-Faccini

**Affiliations:** 1 Graduate Program in Genetics and Molecular Biology, Institute of Biosciences, Universidade Federal do Rio Grande do Sul (UFRGS), Av. Bento Gonçalves, Porto Alegre, 95000, CEP, 91501-970 RS Brazil; 2 Medical Genetics Service, Instituto Nacional de Ciência e Tecnologia de Genética Médica Populacional (INaGeMP), Hospital de Clínicas de Porto Alegre, Porto Alegre, RS Brazil

**Keywords:** Genetic disorders, Congenital anomalies, Public health, Epidemiology, Nigeria

## Abstract

Nigeria, with its rich cultural and genetic diversity, faces a largely undocumented burden of genetic disorders and congenital anomalies. Understanding this burden is vital for effective healthcare planning and interventions. The aim of this study was to provide a comprehensive overview of the prevalence and geographical distribution of genetic disorders and congenital anomalies in Nigeria based on published evidence. A systematic scoping review was conducted across seven databases using tailored search strategies. Screening was performed in two stages: (1) title and abstract and (2) full text- prior to data extraction and analysis. Prevalence figures were recalculated and standardized as a percentage of live births or individuals. Seventy-eight studies met the inclusion criteria, of which 27 reported at least one genetic disorder. Hemoglobinopathies–particularly sickle cell disease (SCD) and sickle cell traits–were the most frequently reported conditions, with prevalence estimates as high as 61%, underscoring Nigeria’s substantial disease burden. Glucose-6-phosphate dehydrogenase (G6PD) deficiency was also commonly reported, with prevalence reaching up to 60% in some populations. Congenital anomalies involving the central nervous system, musculoskeletal system, cardiovascular system, and genitourinary tract were frequently described, with reported prevalence estimates of up to 13%, 8.8%, 3% and 6.7%, respectively. Geographically, studies were disproportionately concentrated in the southern regions, while northern areas–particularly the Northeast–were markedly underrepresented. Significant regional and condition-specific knowledge gaps persist regarding genetic disorders and congenital anomalies in Nigeria, especially in the North. Strengthening surveillance systems, expanding regionally representative research, and implementing targeted public health interventions are critical to inform equitable healthcare planning and reduce disease burden.

**Supplementary Information:**

The online version contains supplementary material available at 10.1007/s12687-026-00876-w.

## Introduction

Genetic disorders have long been a subject of scientific inquiry, evolving from the early recognition of inherited traits to the modern mapping of genes and chromosomal abnormalities associated with both monogenic and polygenic diseases (Karczewski et al. 2020). Alkaptonuria, the first recognized genetic disorder described in the early 20th century, laid the foundation for understanding inborn errors of metabolism (Speicher et al. 2009). Since then, technological advances such as the polymerase chain reaction (PCR) and next-generation sequencing (NGS) have transformed the field, enabling more precise diagnosis and discovery (Heather and Chain 2016; Mardis 2017; Gorzynski et al. 2022).

Congenital anomalies (CAs), which are structural or functional anomalies present at birth, are significant contributors to neonatal morbidity and mortality. These conditions can lead to physical disabilities, developmental delays, or life-threatening complications (Broughan et al. 2024; WHO 2023). Globally, CAs are responsible for approximately 240,000 neonatal deaths annually, with an additional 170,000 deaths occurring between one month and five years of age (Dong et al. 2024; Li et al. 2025). The burden disproportionately affects low- and middle-income countries (LMICs), where up to 90% of severe CAs occur (Sitkin and Farmer 2016; Perin et al. 2023).

Nigeria, Africa’s most populous nation, is home to over 220 million people, comprising more than 370 ethnic groups and over 500 languages. The country is administratively divided into 36 states and the Federal Capital Territory, and further grouped into six geopolitical zones (Joshi et al. 2023). Its vast genetic and cultural diversity presents both unique challenges and opportunities for understanding genetic disorders and congenital anomalies (Joshi et al. 2023). Nigeria also bears one of the highest burdens of sickle cell disease (SCD) globally, with an estimated 1%−3% of the population affected (Nwabuko et al. 2022).

As a country, Nigeria continues to face significant public health challenges, including high mortality rates, with life expectancy estimated at 62.1 years for males and 64.8 years for females in 2021–a modest improvement from 2000 (Adeyemo et al. 2018). The population is growing at 2.1% annually (WHO 2023), with approximately half residing in rural areas, although rural-urban migration is increasing due to socioeconomic factors (Adeyemo et al. 2018). Previous reports have highlighted common genetic disorders such as SCD, Glucose-6-phosphate dehydrogenase (G6PD) deficiency, and albinism, yet a comprehensive national picture of genetic disease burden remains elusive. This paucity of data is compounded by the absence of newborn screening programs, limited diagnostic capacity, and the lack of national registries.

With respect to genetic services, there remains a shortage of both facilities and trained personnel across the country, with advanced services concentrated in cities such as Lagos and the Federal Capital Territory. The earliest medical genetics service was established in the 1970 s in Ibadan, offering clinical and cytogenetic services under the guidance of a paediatric clinical geneticist and a cytogeneticist (Adeyemo et al. 2018). This laid the foundation for subsequent developments in genetic services and research within Nigeria. However, due to scarcity of trained medical geneticists and genetic counsellors, most patients with genetic disorders are managed by paediatricians or other specialists. Although there has been an increase in private and tertiary institutions offering prenatal diagnostic services, such facilities are not widely accessible. Prenatal screening for anomalies has improved detection rates in the past decade, yet limitations in foetal care and uneven access remain major barriers to nationwide uptake (Akinmoladun 2023).

The limited implementation of molecular testing in Nigeria also restricts opportunities for hands-on training in medical genetics. Currently, there are no structured residency, postgraduate programs, or accredited training centers dedicated to clinical genetics in the country, compelling many prospective trainees to pursue advanced training abroad. Although molecular testing is limited, some institutions, such as the Nigerian Institute of Medical Research (NIMR) and the DNA Learning Center Nigeria, offer workshops in molecular testing and hands-on genomic training. Research-focused ethics training for genetic and genomic research is being developed (Gordon et al. 2025).

Congenital anomalies are well documented and contribute substantially to childhood morbidity and mortality. The most frequently affected systems include the musculoskeletal, central nervous, and cardiovascular systems (Famuyiwa 2020; Boma and Tamunoiyowuna 2021). Reported prevalences range from 2.8% to over 15.9%, likely reflecting underreporting (Chimah et al. 2022). Contributing factors include maternal health conditions such as diabetes, genetic predisposition, consanguinity, environmental exposures (including pesticides, alcohol, and certain herbal remedies), nutritional deficiencies, and socioeconomic determinants (Adeboye et al. 2016; Yakubu 2019). These limitations underscore the urgent need for comprehensive data synthesis to inform healthcare policy and service planning.

Despite the high prevalence and clinical significance of these conditions, no prior comprehensive scoping review has synthesized data on the prevalence and distribution of genetic disorders and congenital anomalies in Nigeria. This study aimed to fill this gap by systematically mapping the types, prevalence, and geographic distribution of genetic disorders and congenital anomalies in Nigeria, assessing patterns relative to global data, and identifying critical research and policy gaps. The ultimate goal was to inform public health strategies and guide future research priorities.

## Methodology

This scoping review was conducted following the Joanna Briggs Institute (JBI) methodology for scoping reviews (Peters et al. 2020) and reported in line with the Preferred Reporting Items for Systematic Reviews and Meta-Analyses Extension for Scoping Reviews (PRISMA-ScR) guidelines (Tricco et al. 2018).

### Search strategy

A comprehensive search strategy was developed to identify studies reporting genetic disorders and congenital anomalies in Nigeria. The search was conducted across five electronic databases–PubMed, Web of Science, Embase, Scopus, and African Index Medicus–as well as two grey literature sources (Livivo and Google Scholar). The PubMed strategy was built using Medical Subject Headings (MeSH) and free-text terms including ‘Nigeria’, ‘Genetic Diseases’, ‘Inborn’, ‘congenital abnormalities’, ‘prevalence’, combined with Boolean operators (AND, OR, NOT). This structure was subsequently adapted for each database in consultation with two independent health science librarians. The full set of search strategies is presented in the Supplementary Material. No restrictions were applied regarding publication year or language, and the final search was completed on 13 November 2025. All citations were imported into the Covidence^®^ systematic review platform (Covidence 2020) for screening and data management.

### Eligibility criteria

The inclusion criteria were defined using the Population-Concept-Context (PCC) framework. The Population comprised individuals residing in Nigeria; the Concept covered genetic disorders and congenital anomalies; and the Context included studies reporting epidemiological data, such as prevalence, incidence, or case identification, in hospital-, community-, or population-based settings.

Eligible studies were those presenting primary data on the prevalence, distribution, or epidemiological characteristics of genetic disorders or congenital anomalies in Nigeria. Studies were included if they provided clear geographical information and reported data derived from observational, cross-sectional, cohort, hospital-based, or population-based designs. In contrast, studies were excluded if they did not focus on the Nigerian population, lacked primary data, were reviews, commentaries, or conference abstracts, or failed to include geographical or prevalence-specific information relevant to Nigeria.

### Study selection

**Fig. 1:**
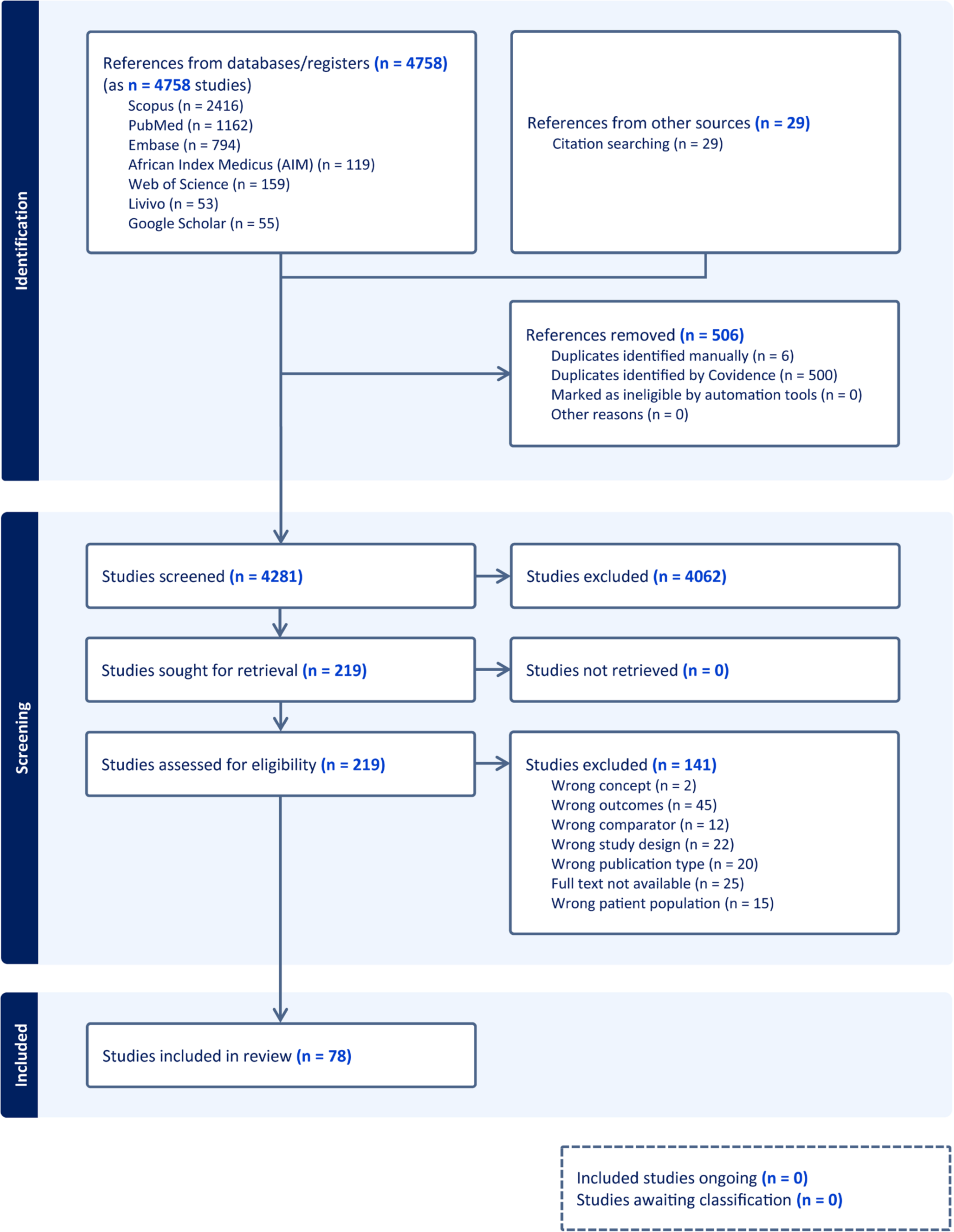
PRISMA flow diagram of different phases of the review

Study selection followed a two-stage screening process. First, titles and abstracts were screened independently by two reviewers (ADB and NFM) to identify potentially relevant articles. Full-text screening was then conducted by the same reviewers, and disagreements were resolved by consensus with input from a third reviewer (LSF). The study selection process is summarized in the PRISMA flow diagram (Fig. 1). Studies that could not be retrieved were further excluded.

### Data extraction

Data extraction was performed using a standardized, pilot-tested form adapted from the Covidence data-extraction template. Extracted information included the author(s) and year of publication, study title, state and geopolitical region, study design, population characteristics, sample size, number of cases, type of genetic disorder or congenital anomaly, and reported prevalence or incidence.

With each reported prevalence, recalculations were performed to confirm the reported values, which were expressed as percentages to one decimal place or three significant figures. In situations where the prevalence was below 0.1%, it was expressed to two decimal places. The prevalence was calculated as shown in the formula below.

Prevalence = $$\left(\frac{Numberofcases}{TotalSampleSize}\right)\times100$$.

The number of cases referred to individuals identified as having a genetic disorder or congenital anomaly in each study, while the Total Sample Size represented the total number of individuals included in the study sample from which those cases were drawn. In studies where the denominator (total sample size) was missing or unclear, the data were excluded from prevalence recalculation to ensure accuracy and comparability across estimates.

### Data synthesis and mapping

Descriptive analyses were performed using R (version 4.3.3). Prevalence estimates were summarized by type of condition, anatomical classification (for congenital anomalies), and geographical distribution across Nigeria’s six geopolitical zones. Geospatial visualization was conducted using QGIS (version 3.16.3) to produce choropleth maps representing the distribution of genetic disorders and congenital anomalies across the country. When multiple studies reported the same condition in a given region, mean prevalence values were computed to produce consolidated regional estimates.

## Results

### Study characteristics

A total of 4787 articles were retrieved from the databases. After removing 506 duplicates, 4,281 titles and abstracts were screened. Of these, 4,062 were excluded because they did not meet the eligibility criteria. The full texts of 219 articles were reviewed, leading to the exclusion of 141 studies, primarily due to irrelevant outcomes (36.2%). Ultimately, 78 studies met the inclusion criteria and were included in the analysis reported (Fig. 1; Supplemental 1).

### Publication trends

**Fig. 2:**
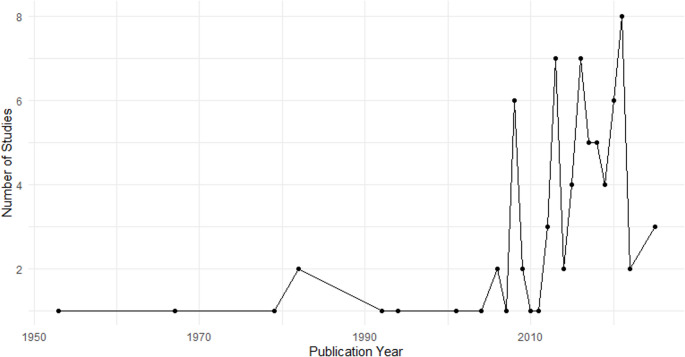
Number of publications in Nigeria on genetic disorders and congenital anomalies per year

Research activity demonstrated an upward trajectory post-2000, peaking in 2021 with eight studies published. From 2016 to 2021, publication output remained steady with an average of five articles yearly, reflecting sustained interest and awareness in genetic disorders and congenital anomalies in Nigeria (Fig. 2).

### Study design

The majority of the studies were cross-sectional observational studies (*n* = 49; 62.8%). Other study designs are presented in Table 1.

### Geographical distribution

**Fig. 3:**
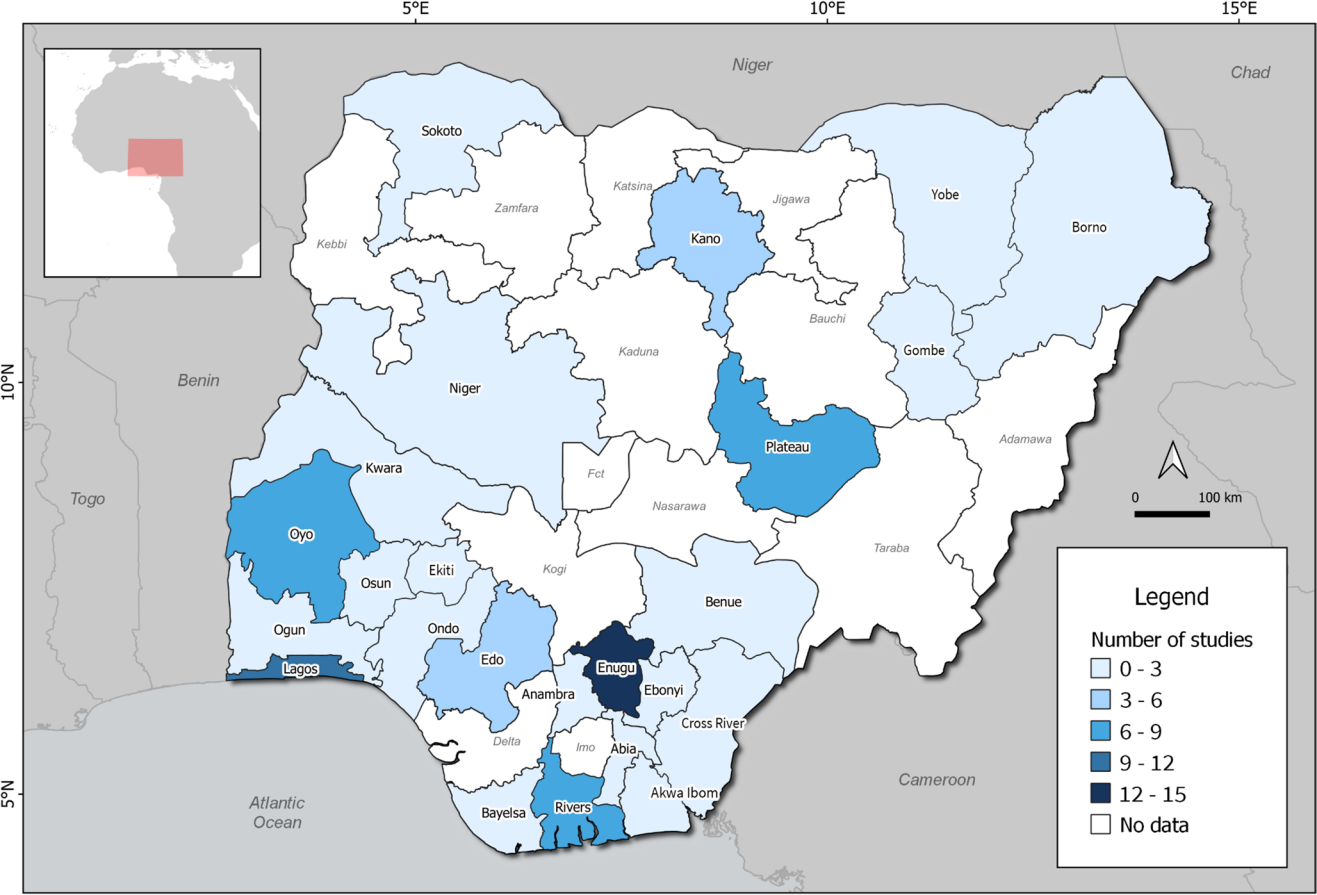
Geographic distribution of publications on genetic disorders and congenital anomalies in Nigeria. Note: Two multicenter studies were included: one covering Kano, Borno, Yobe, Gombe, Lagos, and Enugu, and another covering Cross River and Akwa Ibom

The included studies spanned 23 of Nigeria’s 36 states and the Federal Capital Territory. Enugu State accounted for the highest number of studies (*n* = 15; 19.2%), followed by Lagos (12; 15.4%) Oyo and Plateau (*n* = 8; 10.3%) (Fig. 3). The geopolitical regions’ distributions are presented on Table 1.

**Table 1:** Study design and geopolitical distribution

Study design	Number of studies	Percentage (%)
Cross-sectional	49	62.8
Retrospective (Hospital-based)	12	15.4
Prospective (Hospital-based)	7	9.0
Cohort	3	3.9
Population-based prospective	1	1.3
Hospital-based observational	2	2.6
Case-control	1	1.3
Other (mixed/ambiguous)	3	3.9
Region		
South-West	27	35.1
South-East	19	24.7
South-South	14	18.2
North-Central	12	15.6
North-West	5	6.5

NB: One (1) study from SMILE Train (Butali et al. 2014) covered all regions except North−Central and South−South

### Genetic disorders

Of the 78 included studies, 27 reported on genetic disorders, primarily hemoglobinopathies and enzymopathies. All prevalence estimates were recalculated and standardized in percentages (Table 2; Fig. 3).

### Glucose-6-phosphate dehydrogenase (G6PD) deficiency

The prevalence ranged from 4.2% to 60.0% across reported studies. The highest prevalence was reported in Enugu (60.0%) (Engwa et al. 2017). Other notable figures included a recent study reporting a prevalence of 40.0% in Plateau (Niandat and Okoli 2025), and 15.3% in Oyo (Williams et al. 2013). Across four regions (North-Central, North-West, South-East, and South-West), the highest prevalence was in the South-East, affecting 60.0% of the study population.

### Hemoglobinopathies (including sickle cell disease and traits)

These were the most extensively studied genetic disorders with widely varied prevalence. In the South-South, the prevalence ranged from 2.0% to 26.3%; in the South-East, from 13.6% to 27.4%; and in the North-Central, from 11.3% to 48.0%. The South-West had a prevalence of carrier traits only, ranging from 20.0% to 24.5%. The highest prevalence was reported in the North-West as 61.8%.

Two studies, Oluwole et al. (Oluwole et al. 2020) and Stephen et al. (Stephen et al. 2018), reported SCD in isolation, with prevalence of 1.0% and 2.7%, respectively. Other studies reported SCD alongside other hemoglobinopathies, and when individualized, prevalence ranged from 0.1% to 7.5% in the North-Central region, and a staggering 51.7% was recorded for SS, SC, and SS + F in the North-West (Saganuwan 2016). Similarly, ranges of 0.1% to 5.8% and 0.1% to 1.6% were reported for South-South and South-East, respectively. All regions recorded consistently high prevalence, except the North-East, for which no direct data were available.

### Other genetic disorders

**Fig. 4:**
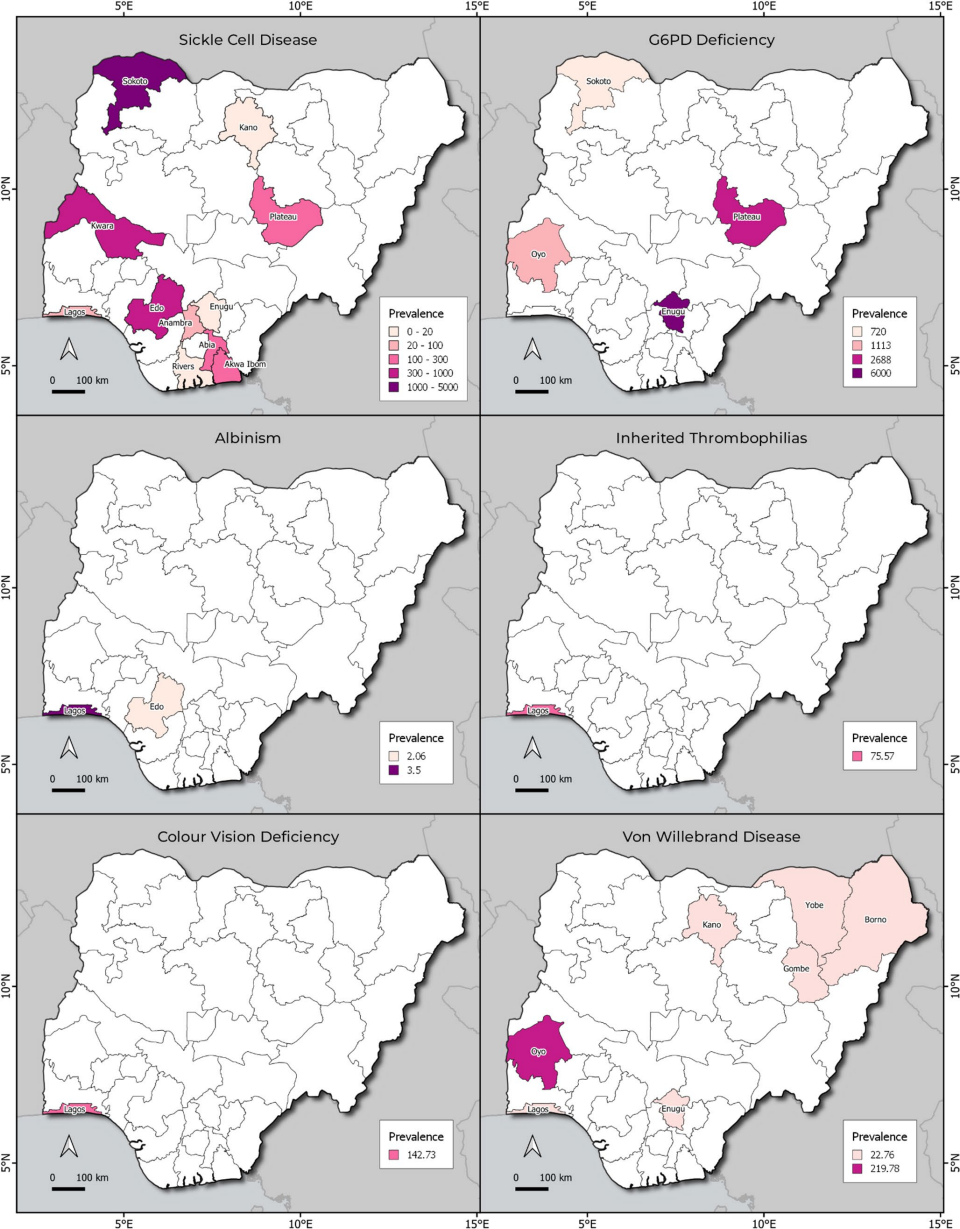
Prevalence of sickle cell disease (SCD), G6PD deficiency, albinism, inherited thrombophilia, color vision deficiency, and von willebrand disease, across Nigerian states

Beyond enzymopathies and hemoglobinopathies, fewer studies have documented less commonly investigated genetic disorders in Nigeria, particularly in the South-West region. Shonde-Adebola et al. (2021) studied Von Willebrand disease and reported a prevalence of 2.2%. Inherited thrombophilia, which increases the risk of abnormal blood clotting, was examined by John-Olabode et al. (John-Olabode et al. 2021), revealing a prevalence of 0.8%. Similarly, Fakorede et al. (Fakorede et al. 2022) investigated inherited color vision deficiency and found a notable prevalence of 2.8%. Furthermore, Barnicot (Barnicot 1952) reported the prevalence of albinism as 0.03% and 0.02% in the South-West and South-South regions, respectively (Fig. 4).

**Table 2:** Genetic disorders, regions and prevalence reported

Genetic Disorder	Study ID	State (Region) *N* Sample	*N* Cases	Prevalence (%)
G6PD Deficiency	Egesie et al.2008	Plateau (North-Central) *N* = 126	26	20.6
	Egesie et al.2013	Plateau (North-Central) *N* = 130	26	20.0
	Niandat and Okoli 2025	Plateau (North-Central) *N* = 100	40	40.0
	Isaac et al.2013	Sokoto (North-West) *N* = 118	12 5	10.2(Moderate) 4.2 (Severe)
	Engwa et al. 2017	Enugu (South-East) *N* = 95	57	60.0
	Ajao 2019	Oyo (South-West) *N* = 1,057	50	4.7
	Gilles et al.1967	Oyo (South-West) *N* = 300	40	13.3
	Williams et al. 2013	Oyo (South-West) *N* = 1,122	172	15.3
Hemoglobinopathies (Including SCD)	Ameen et al.2016	Kwara (North-Central) *N* = 440	144 AS 34 AC 27 SS 6 SC	32.7 7.7 6.1 1.4
	Egesie et al.2013	Plateau (North-Central) *N* = 130	27 AS	20.8
	Ezenwosu et al.2021	North-Central *N* = 10,167	3 AC 1,906 AS 1 SC 5 SS	0.03 18.7 0.01 0.05
	Fleming et al.1979	Kano (North-Central) *N* = 2,742	793 AS 19 AC 1 SC 4 SS	28.9 0.7 0.04 0.1
	Stephen et al. 2018	Plateau (North-Central) *N* = 3,047	82 SCD	2.7
	Saganuwan 2016	Sokoto (North-West) *N* = 319	32 AS 102 SS 56 SC 7 SS + F	10.0 32.0 17.6 2.19
	Burnham-Marusich et al.2016	Enugu (South-East) *N* = 5,545	746 AS 4 SS 4 AC	13.5 0.07 0.07
	Nnaji et al.2013	Anambra (South-East) *N* = 424	112 AS 4 SS	26.4 0.9
	Nwabuko et al. 2022	Abia (South-East) *N* = 8,457	1876 AS 136 SS 2 SC	22.2 1.6 0.02
	Abhulimhen-Iyoha et al.2013	Edo (South-South) *N* = 589	118 AS 3 AC 34 SS	20.0 0.05 5.8
	Nwabuko 2015	Rivers (South-South) *N* = 35,976	7,109 AS 52 SS	19.8 0.1
	Odunvbun et al. 2008	Edo (South-South) *N* = 644	18 SS 1 SC 133 AS 7 AC	2.8 0.02 20.6 1.1
	Umoh et al. 2010	Akwa Ibom (South-South) *N* = 8,097	1580 AS 121 SS 16 AC 4 SC	19.5 1.5 0.2 0.05
	Akinbodewa et al. 2022	Ondo (South-West) *N* = 102	4 AC 21 AS	4.0 21.0
	Gilles et al.1967	Oyo (South-West) *N* = 300	20 AC 40 AS	6.7 13.3
	Oluwole et al. 2020	Lagos (South-West) *N* = 250	1 SS 1 CC 1 SC 53 AS 9 AC	0.4 0.4 0.4 21.2 3.6
Von Willebrand Disease	Butali et al. 2014	Kano (North-West), Borno, Yobe, and Gombe (North-East), Lagos (South-West), Enugu (South-East) *N* = 2,197	5	0.23
	Shonde-Adebola et al. 2021	Oyo (South-West) *N* = 182	4	2.2
Inherited Thrombophilias	John-Olabode et al. 2021	Lagos (South-West) *N* = 397	3	0.8
Colour Vision Deficiency	Fakorede et al. 2022	Lagos (South-West) *N* = 1,191	18 16	1.5 (Deutan CVD) 1.3 (Protan CVD)
Albinism	Barnicot 1953	Lagos (South-West) *N* = 14,292 Edo (South-South) *N* = 4,862	5 1	0.03 0.02

### Congenital anomalies based on the anatomical system

A detailed description of the studies involving structural congenital anomalies is shown in Supplemental 1 and 2.

### Central nervous system (CNS) and neural tube defects (NTDs)

CNS anomalies were consistently among the most reported, with neural tube defects particularly prevalent. Common conditions included spina bifida, anencephaly, hydrocephalus, and microcephaly. The highest prevalence recorded was in Lagos with 10.6% for microcephaly (Olusanya 2012) followed by Niger, 2.0% (specific CNS anomalies were not described) (Adeboye et al. 2016). Another study reported a prevalence of 0.1% for anencephaly in the South-West region.

### Cardiovascular system (congenital heart defects, CHD)

Congenital heart defects (CHDS) commonly reported were ventricular septal defects (VSD), atrial septal defects (ASD), patent ductus arteriosus (PDA), and Tetralogy of Fallot (ToF). The reported prevalence ranged from < 0.01% to 3.0% in Lagos (Animasahun et al. 2017) followed by Plateau with 2.9% (Ige et al. 2021). Other notable prevalence included Rivers with 1.4% (Otaigbe and Tabansi 2014), Edo with 1.4% (Sadoh et al. 2021), and Oyo with 1.0% (Ajao and Adeoye 2019). In Lagos, two studies evaluated the prevalence of ToF and reported a prevalence of 0.05% (Animasahun et al. 2015, 2016). Population-based screening studies, such as Ekure et al. (Ekure et al. 2020), further demonstrated substantial diagnostic gaps in the detection of congenital heart disease among Nigerian school-aged children.

### Gastrointestinal (GI) anomalies

GI anomalies reported included imperforate anus (0.03% in Enugu, 0.04% in Rivers, and 0.02% in Cross River/Akwa Ibom), omphalocele (0.02% in Enugu, and 0.01% in Ondo), gastroschisis (0.02% in Enugu, and 10.0% in Ondo), and esophageal atresia with tracheoesophageal fistula (1.0% Oyo, and 0.07% in Rivers). Malrotation and diaphragmatic hernia were also reported. The highest prevalence estimates were reported in Niger, Benue, Edo, Oyo, and Osun, at 5.8%, 3.2%, 2.0%, 1.5%, and 1.1%, respectively. Obu et al. (2012) reported a prevalence of anorectal malformation of 0.2%.

### Musculoskeletal/limb defects (MSD)

The highest prevalence estimates reported for MSD were in Bayelsa with 13.1% (6.6% postaxial polydactyly, which was considered a minor anomaly) (Oyinbo et al. 2009), 3.4% in Osun (Bakare et al. 2009), 2.3% in Benue (Eseigbe et al. 2022), and 2.0% in Niger (Adeboye et al. 2016). Clubfoot (talipes equinovarus), was also reported to have the highest prevalence of 0.8% in Bayelsa (Oyinbo et al. 2009), and 0.6% in Oyo (Ajao and Adeoye 2019). Limb reduction defects, polydactyly and syndactyly, arthrogryposis, and congenital hip dislocation were also reported. Some MSDs were often observed alongside neural tube defects in some studies (Ekwochi et al. 2018; Adeboye et al. 2016; Sunday-Adeoye et al. 2007; Ajao and Adeoye 2019; Ekanem et al. 2008; Chukwubuike et al. 2020; Abbey et al. 2017a; Oluwafemi and Abiodun 2019a; Akinmoladun et al. 2018). Amniotic band sequence had a prevalence of 3.0% in Bayelsa (Oyinbo et al. 2009).

### Genitourinary anomalies

Frequently reported was hypospadias with a prevalence of 0.5% in Osun, and less than 0.01% in Ebonyi, Enugu and Oyo. Other related conditions reported were cryptorchidism (undescended testes), posterior urethral valves, bladder exstrophy, ambiguous genitalia, and polycystic kidney disease. The highest overall prevalence reported was in Enugu with 6.7% and 3.5% (Ozoemena and Mbah 2006; Anyimba et al. 2025), followed by Osun, 1.4% (Bakare et al. 2009) and Niger, 1.2% (Adeboye et al. 2016). Notably, the prevalence of cryptorchidism in Osun State was 2.5% (Adeoti et al. 2004).

### Craniofacial anomalies

Orofacial clefts, including cleft lip with or without cleft palate, with prevalence ranging from ~ 2.5% (Cross River/Akwa Ibom) to 0.8% and cleft lip/palate 0.5% (Bayelsa) were widespread. While the highest prevalence recorded was in Bayelsa at 1.3% (Oyinbo et al. 2009), a multicenter dataset reported 0.05% for orofacial clefts only (Butali et al. 2014).

### Ocular (eye) anomalies

Ocular anomalies were seldom reported but included: microphthalmia (0.1% in Kano) (Lawan 2008), congenital cataract (0.3% in Ogun) (Bodunde and Ajibode 2006). Congenital glaucoma and dermoid cysts were also noted. The highest prevalence were reported by Adekoya et al. (Adekoya et al. 2015) in Lagos with 9.7%, Lawan (Lawan 2008) in Kano with 1.6% and Bodunde et al. (Bodunde and Ajibode 2006) in Ogun with 0.7%.

### Skin/integumentary anomalies

Five studies reported this condition and one of which combined head and neck leading to a prevalence of 1.5% (Adeboye et al. 2016). Other reported anomalies included hypopigmentation and hyperpigmentation of the face and neck, widely spaced nipples, slanting palpebral fissure, single palmar creases, redundant skin at back of neck.

### Pulmonary/respiratory anomalies

Rarely reported with four studies reporting this set of conditions. The anomalies reported included diaphragmatic hernia, laryngomalacia and nasal anomalies. The prevalence ranged from 0.02% to 0.1%, with reports from Oyo, Lagos, Ogun, and Cross River/Akwa Ibom.

### Chromosomal and syndromic conditions

A total of 20 studies noted chromosomal anomalies or multi-system syndromes and included trisomies of which Down syndrome was the most reported with the highest prevalence of 0.5% in Oyo (Ajao and Adeoye 2019). Other reported syndromes included Patau, Turner, Edwards, Pierre Robin, CATCH 22, Beckwith-Wiedemann and Prune Belly syndromes.

### Others/multiple

Some studies reported complex multifactorial or multiple anomalies not associated with a syndrome, having the highest prevalence reported in Oyo (0.8%) (Akinmoladun et al. 2018), Rivers (0.3%) (Abbey et al. 2017a), and Kano (0.2%) (Anyanwu et al. 2015). Other conditions such as hypothyroidism in Rivers and macrosomia in Osun were reported (Abbey et al. 2017a; Bakare et al. 2009).

### Regional patterns

The types of anomalies reported varied by region; the southern regions had more studies and broader anomaly spectra (Supplemental 2). The key patterns included:

#### South-East (20 studies)

 CNS/neural tube defects, orofacial clefts, congenital heart defects, genitourinary anomalies, gastrointestinal defects, ocular defects, and musculoskeletal malformations were reported. The most documented anomalies with at least 4 studies were CNS, CHD, GI, MSD, genitourinary, and craniofacial anomalies. This region also accounted for the highest prevalence of genitourinary and craniofacial anomalies.

#### South-West (28 studies)

Commonly reported anomalies included genitourinary defects, gastrointestinal defects, neural tube defects, and musculoskeletal anomalies. Studies here accounted for many of the very high prevalence such as clubfoot in Oyo. This region also reported at least 4 publications for all anomalies excluding ocular, integumentary, and pulmonary anomalies.

#### South-South (14 studies)

This region featured mainly CNS, CHD, MSD and orofacial clefts, along with some GI and genitourinary anomalies. The large Rivers State study found the highest overall prevalence of congenital anomalies in Nigeria (Abbey et al. 2017a). The South-South region also had the highest documented chromosomal anomalies.

#### North-Central (14 studies)

Cases of congenital anomalies were reported across CNS, CHD, GI, MSD, and genitourinary with at least two reports in each of the affected systems. Notable findings included gastrointestinal malformations (omphalocele, diaphragmatic hernia) and isolated findings like congenital glaucoma (Kwara) or ear anomalies.

#### North-West (4 studies)

Very limited data were available for this region and reported cases included ocular defects (microphthalmia in Kano) and a few musculoskeletal defects.

## Discussion

This scoping review represents the most comprehensive synthesis to date of genetic disorders and congenital anomalies in Nigeria. The findings reveal a substantial disease burden relative to global estimates, significant regional disparities, and notable gaps in data coverage, particularly in the Northern regions of the country. Notably, publication output has risen steadily since the early 2000s, peaking in 2021. This trend likely reflects increased global attention to congenital anomalies and genetic disorders in LMICs, progressive improvements in diagnostic capacity within the Nigerian tertiary hospitals, and the expanding research culture associated with teaching hospitals and postgraduate training. Continental and sub-continental initiatives such as H3Africa and Sub-Saharan Congenital Anomalies Network (sSCAN) have further strengthened research infrastructure, data governance, collaborative networks and campaigns on food fortifications, awareness, and other mitigating strategies. The growing availability of open access publishing platforms and gradual introduction of digitalized hospital records has lowered barriers to research dissemination (Olukorode et al. 2024). Together, these developments likely underpin the sustained rise in scientific output observed in these fields.

### Genetic disorders

This review confirms that hemoglobinopathies, especially sickle cell disease (SCD), remain the most prevalent and extensively studied genetic disorders in Nigeria. The highest prevalence reported for SCD, and its traits was in the North-West, reinforcing Nigeria’s position as the global epicenter for SCD (Nwabuko et al. 2022). Contributing factors to this prevalence are likely high frequencies of endogamy and homogamy, reported at 78% and 90% respectively (Okigbo 2023; Isiugo-Abanihe and Fayehun 2017). SCD continues to pose significant public health challenges, contributing heavily to morbidity, mortality, and healthcare system strain (Dilli et al. 2024). The implementation of premarital genetic screening programs, while helpful in some contexts, has raised ethical concerns around autonomy, discrimination, and stigma. A growing body of evidence supports shifting focus toward early-life or universal carrier screening combined with genetic counselling, which may provide more equitable and effective preventive strategies (Ezugwu et al. 2019).

Similarly, the persistent burden of G6PD deficiency highlights the evolutionary trade-off between malaria resistance and hemolytic risks, a well-documented phenomenon in sub-Saharan Africa (Leslie et al. 2010; Ruwende et al. 1995; Santana et al. 2013; Luzzatto et al. 2020). The hemolytic risk poses a grave consequence, especially among children (Uyoga et al. 2015; Luzzatto 2015). Despite its high prevalence, G6PD deficiency remains underdiagnosed, with limited public health efforts directed toward screening or management, especially in regions with malaria co-endemicity, such as the North-Central and North-West shown in this study.

Comparatively, genetic disorders such as Von Willebrand disease, thrombophilias, color vision deficiency, and albinism were infrequently reported, with the majority of cases found in the South-West. Von Willebrand disease which is the most common inherited bleeding disorder remains underdiagnosed mainly due to limited awareness, diagnostic tools, and treatment access (Nigeria Health Watch 2024; Ezigbo et al. 2020). There is a higher prevalence in youths than is obtainable in global averages (Shonde-Adebola et al. 2021). Thromobophilia on the other hand, was found to have a lower prevalence in Nigeria and this could be the effect of a small sampling size which was restricted to a particular region thus, might not be representative of the entire population (John-Olabode et al. 2021). Albinism has been estimated to affect about 2 million people in Nigeria and occurs as a result of mutations that affect the production and availability of melanin, leading to little or no pigmentation for skin, hair and eyes (Federico and Krishnamurthy 2025). Asides from the clinical implication of this condition, affected persons are posed with other delipidating threats such as cultural myths, misconceptions and ignorance presenting a greater threat and more importantly, pointing to the need for interventions to reduce the risk people with albinism are faced with (Ejilola 2025). Overall, there are scarce reports on other monogenic diseases and inborn error of metabolism which has in recent years gained global audience and advances. One study which was not included in this study because it was not epidemiological in design reported the presence of Machado-Joseph’s disease in Nigeria (Ogun et al. 2015). The rarity of some of these conditions and the diagnostic odyssey associated with them could reduce the potential of being reported (Baynam et al. 2016; Bauskis et al. 2022). This reflects significant diagnostic inequities, with better-equipped urban centers producing more diverse research outputs.

### Congenital anomalies (CAs)

Neural tube defects (NTDs) were the most commonly reported congenital anomalies, with anencephaly and spina bifida accounting for a substantial proportion of cases in Nigeria. CAs of CNS have been reported to account for approximately 0.2%−13% of live births (Oluwafemi and Abiodun, 2019a; Olusanya 2012), and in some southern regions, they constitute between 27% and 41% of all documented congenital anomalies (Abbey et al. 2017b; Oluwafemi and Abiodun 2019b). Notably, several prevalence estimates reported in this review from the North, South-East and South-South regions exceed global estimates of 1–5 per 1,000 live births with low- and middle-income countries (LMICs) known to bear a disproportionately higher burden.

Likely contributing factors could be low folic acid intake and folic acid supplementation programmes, poor maternal nutrition, limited access to prenatal care, and education (Okon et al. 2020). Another factor often overlooked is the impact of environmental exposures, such as gas flaring, particularly in the South which has been associated with NTDs (Gaughan et al. 2023). Emerging but inconclusive evidence also points to possible associations between HIV treatment (dolutegravir) and increased NTD risks, although recent studies mitigate some of these concerns (Zash et al. 2019; Pereira et al. 2021). Together, these suggest a need for enhanced maternal health initiatives, including folate supplementation and better access to prenatal diagnostic services at an affordable cost, to reduce the incidence of NTDs.

Musculoskeletal anomalies, particularly clubfoot, and limb malformations, showed elevated prevalences in Oyo and Bayelsa exceeding global prevalence (1.18 per 1,000) and LMICs estimates as Africa (1.31) and Southeast Asia (1.80) (Smythe et al. 2023). The frequent co-occurrence of musculoskeletal anomalies with NTDs especially in the South-East and South-South regions suggests shared developmental pathways, potentially influenced by both genetic predispositions and environmental teratogens (Stoll et al. 2011). The reported prevalence may be a true reflection of an improved reporting system or ease of diagnosis. It is however possible that regional environmental teratogens such as gas flaring (Gaughan et al. 2023), and site of study (mostly hospital-based), played a role in the elevated frequencies observed.

Craniofacial anomalies, especially orofacial clefts, were widespread but mostly within global prevalence ranges. Historically, children born with such defects faced significant stigma, neglect, or infanticide due to cultural beliefs (Oginni et al. 2010). Interventions from non-governmental organizations, such as *Smile Train*, have significantly improved outcomes through enhanced access to surgery and multidisciplinary care since 2006 (Butali and Adeyemo 2011).

Congenital heart defects (CHDs) were frequently reported in southern Nigeria, with some prevalences surpassing global averages of 8 per 1,000 live births (Hasan et al. 2023; van der Linde et al. 2011). The high level of undiagnosed CHD among school children, as reported by Ekure et al. (Ekure et al. 2020) in a megacity, Lagos, reveals gaps in the healthcare system, of which advanced or urban cities in Nigeria are not exempt. The regional prevalence could be the result of better diagnostic capacity in urban centers, environmental pollution, especially particulate matter and hydrocarbons from increased industrial activities, which have been documented as teratogens associated with CHDs such as ToF and observed in this study at a prevalence level higher than global prevalence (3–4 per 10,000) (Llamosas-Falcón et al. 2019; Bailliard and Anderson 2009; Liu et al. 2019). Overall, echocardiographic screening for school children, proposed by Ekure et al. (Ekure et al. 2020), could provide an additional opportunity for early diagnosis and treatment of CHD, thereby reducing the prevalence of first diagnosis in adulthood.

Ocular anomalies were found to be highest in the South-West and North-West, and could be associated with the use of pesticides in agrarian settlements, as well as biomass smoke, which significantly affects regions in Nigeria, mainly in rural areas (Nwankwo et al. 2018). Pesticides, such as the combination of chlorpyrifos and cypermethrin, have been shown to impact developing chick embryos and may play a role in the development of eyes in humans. This is due to the alteration in Sonic Hedgehog gene expression, which is pivotal in the proper patterning of the eye in association with *PAX6* gene (Sharma et al. 2025).

Gastrointestinal anomalies were frequently reported at prevalence similar to or exceeding global figures (15.5 per 10,000), consistent with other LMIC trends (Global PaedSurg Research Collaboration 2021; Sitkin et al. 2015). Underlying causes may include micronutrient deficiencies, young maternal age and infections, and use of herbal medications, which are prevalent in Nigerian settings (Congenital Disorders n.d.; Goel et al. 2006).

Genitourinary anomalies, particularly hypospadias, exhibited higher prevalence than the global prevalence (11.3 per 10,000) but were comparable to regional prevalence in North America, Europe, Asia, and South America (Yu et al. 2019). Possible drivers of this condition include maternal malnutrition, chronic lead exposure, pesticide use, and perinatal infections (Bakare et al. 2002; Aboyeji and Nwabuisi 2003; Droller 1996).

Chromosomal abnormalities, particularly Down syndrome, were reported primarily in the South-West, where advanced prenatal diagnostic services are more accessible. The most common severe aneuploidy at the time of birth is Down syndrome (Arbuzova et al. 2002) which can be associated with intellectual and motor developmental impairment, facial dysmorphic features and congenital anomalies, including congenital heart conditions in some cases (Verstegen and Kusters 2020). Notwithstanding, the high prevalence observed in a recent study by Ajao and Adeoye (Ajao and Adeoye 2019), reporting at least four times greater than the global prevalence of 1 in 1,000 (Grimm et al. 2021) may reflect both true prevalence and improved detection. A critical factor that has been shown to predispose to chromosomal anomalies is advanced maternal age (Kim et al. 2013; Elmerdahl Frederiksen et al. 2024; Orazulike et al. 2015). In recent years, there have been reports of a significant percentage of Nigerian pregnancies occurring at advanced maternal age with figures as high as 10.9% in a study (Solanke et al. 2020). This was associated with socio-demographic factors including higher maternal education, age at first marriage, modern contraceptive use, remarriage status, high community literacy level and a high proportion of women who never used modern contraceptives (Solanke et al. 2019; Akinyemi et al. 2015).

### Regional variations and data gaps

This review highlights stark regional disparities in the prevalence of genetic disorders and congenital anomalies across Nigeria. The South-West and South-East regions dominate in research output and anomaly diversity, likely due to better healthcare infrastructure and research capacity, and diagnostic services. In contrast, the North-West and North-Central regions are underrepresented, with sparse data and narrower anomaly spectra. Furthermore, the North-East is almost entirely absent, reflecting the compounding impact of armed conflict, instability, poor access to healthcare, and a lack of research funding. These patterns align with global trends, in which LMICs with sociopolitical instability and weak healthcare systems suffer from major data gaps (Abdul-Rahman et al. 2023).

### Public health implications

The findings from this study underscore the pressing need for targeted public health interventions in Nigeria, such as strengthening maternal and child health services, particularly in underserved regions, including those with high burdens of genetic and congenital anomalies. Establishing birth registries would greatly improve population-level data collection and surveillance. Given the high prevalence of SCD and G6PD deficiency, Nigeria would greatly benefit from comprehensive screening programs (including prenatal screening and counseling services) and public health campaigns to strengthen maternal and child health services. Further promoting folic acid supplementation programs would help prevent NTDs. Enforcing environmental health regulations, particularly those that control exposure to gas flaring, pesticides, and heavy metals, which contribute to congenital anomalies, would also be important.

In the global pattern context, Nigeria faces a disproportionately high burden of hemoglobinopathies, NTDs, and genitourinary anomalies, compounded by limited healthcare access, environmental exposures, and sociocultural factors. Addressing these disparities will require both national commitment and international collaboration, adopting lessons from countries with successful congenital anomaly prevention programs.

## Limitations

The quality of data reported in this review synthesizes publicly available data, which is inherently affected by the limited availability of genetic services, specialized healthcare, and diagnostic infrastructure and laboratories in Nigeria. There is also the impact of sociocultural factors within the African context, where, for instance, children with certain congenital anomalies are regarded as having a spiritual undertone, thereby reducing the likelihood of seeking medical help (Getnet et al. 2025; Emordi and Osifo 2018). This is also reflected in the reliance on traditional healers and birth attendants, which reduces the number of cases presented at hospitals, the primary source for most of the articles reviewed.

As with most scoping reviews, this study has several limitations. First, the analysis relied primarily on hospital-based data, which may disproportionately reflect more severe cases and underestimate conditions that result in fetal losses, stillbirths, or undiagnosed anomalies in community settings. Second, the geographical distribution of studies was highly uneven, with the North-East completely unrepresented and limited data from the North-West and North-Central. This reflects broader disparities in healthcare infrastructure, research capacity, and regional instability. Furthermore, selection bias may have occurred due to the overrepresentation of studies from better-resourced southern states, which have greater diagnostic capabilities and academic institutions. As a scoping review, the study did not conduct a formal risk of bias assessment or meta-analysis, limiting the ability to draw causal inferences or compute pooled estimates.

## Conclusion

This scoping review highlights that Nigeria bears a disproportionately high burden of genetic disorders and congenital anomalies, with significant regional disparities in prevalence, data availability and research output.

Hemoglobinopathies, particularly sickle cell disease, and enzymopathies like G6PD deficiency, are the most common genetic disorders, posing substantial challenges to the Nigerian healthcare system. Similarly, congenital anomalies-including neural tube defects, musculoskeletal malformations, congenital heart defects, and genitourinary anomalies-were reported at frequencies exceeding global averages, especially in regions with better diagnostic facilities.

In stark contrast, northern regions of Nigeria remain critically underrepresented, reflecting both healthcare inequities and sociopolitical barriers. These findings underscore the urgent need for a national birth defect and genetic surveillance system, expanded genetic counseling services, and enhanced maternal and child health policies in underserved regions.

## Supplementary Information

Below is the link to the electronic supplementary material.

Supplementary Material 1

## Data Availability

No datasets were generated or analysed during the current study.
