# Supplementary material for: Genetic disorders and congenital anomalies in Nigeria: a scoping review
Source: J Community Genet. 2026 Apr 9;17(2):47. doi: 10.1007/s12687-026-00876-w (PMC13062147; doi:10.1007/s12687-026-00876-w)
Supplement: Supplementary file 1 — Supplementary Material 1 [file 12687_2026_876_MOESM1_ESM.docx]

**GENETIC DISORDERS AND CONGENITAL ANOMALIES IN NIGERIA: A SCOPING REVIEW**

Journal of Community Genetics

Ayoade Desmond Babalola^1^ (ORCID ID: 0000-0003-0517-255X), Nitza Ferreia Muniz^1^ (ORCID ID: 0000-0002-7170-3315), Lavinia Schuler-Faccini^1,2^ (ORCID ID: 0000-0002-2428-0460)

1. Graduate Program in Genetics and Molecular Biology, Institute of Biosciences, Universidade Federal do Rio Grande do Sul (UFRGS), Av. Bento Gonçalves, 95000, Porto Alegre, RS CEP, 91501-970, Brazil
2. Medical Genetics Service, Instituto Nacional de Ciência e Tecnologia de Genética Médica Populacional (INaGeMP), Hospital de Clínicas de Porto Alegre, Porto Alegre, RS, Brazil

**Supplementary Files**

**Search strategy**

**PubMed** **-** <https://pubmed.ncbi.nlm.nih.gov/>

**#1**  "Nigeria"[Mesh] OR Nigeria*

**#2**"Genetic Diseases, Inborn"[Mesh] OR “genetic disease*" OR "hereditary disease*" OR "genetic disorder*" OR "single-gene defect*" OR "single gene defect*" OR "congenital disorder*" OR "inherited disease*" OR "inherited disorder*" OR "genetic syndrome*" OR "genetic defect*" OR "hereditary affliction" OR "congenital abnormalities"[Mesh] OR "birth defect*" OR "congenital anomal*" OR "congenital malformation*" OR "fetal malformation*"

**#3** "prevalence"[MeSH Terms] OR "epidemiology"[Subheading] OR prevalence[Text Word]

**#4** #1 AND #2 AND #3

**Web of Science -** https://www.webofscience.com/wos/woscc/basic-search

**#1** ALL=(nigeria*)

**#2** ALL=("genetic disease*" OR "hereditary disease*" OR "genetic disorder*" OR "single-gene defect*" OR "single gene defect*" OR "congenital disorder*" OR "inherited disease*" OR "inherited disorder*" OR "genetic syndrome*" OR "genetic defect*" OR "hereditary affliction" OR "congenital abnormalit*" OR "birth defect*" OR "congenital anomal*" OR "congenital malformation*" OR "fetal malformation*")

**#3** ALL=(prevalence OR epidemiology)

**#4** #1 AND #2 AND #3

**Scopus -** https://www.scopus.com/search/form.uri?display=basic#basic

**#1** ALL (nigeria*)

**#2** ALL ("genetic disease*" OR "hereditary disease*" OR "genetic disorder*" OR "single-gene defect*" OR "single gene defect*" OR "congenital disorder*" OR "inherited disease*" OR "inherited disorder*" OR "genetic syndrome*" OR "genetic defect*" OR "hereditary affliction" OR "congenital abnormalit*" OR "birth defect*" OR "congenital anomal*" OR "congenital malformation*" OR "fetal malformation*"

**#3** TITLE-ABS-KEY (prevalence OR epidemiology)

**#4** #1 AND #2 AND #3

**Embase -** https://www.embase.com/search/quick

**#1** 'nigeria'/exp OR nigeria*:ti,ab,kw

**#2** 'genetic disorder'/exp OR 'genetic disease*' OR 'hereditary disease*' OR 'genetic disorder*' OR 'single-gene defect*' OR 'single gene defect*' OR 'congenital disorder*' OR 'inherited disease*' OR 'inherited disorder*' OR 'genetic syndrome*' OR 'genetic defect*' OR 'hereditary affliction' OR 'congenital disorder'/exp OR 'birth defect*' OR 'congenital anomal*' OR 'congenital malformation*' OR 'fetal malformation*'

**#3** 'prevalence'/exp OR prevalence:ti,ab,kw OR 'epidemiology'/exp OR epidemiology:ti,ab,kw

**#4** #1 AND #2 AND #3

**#5** [embase]/lim NOT ([embase]/lim AND [medline]/lim)

**#6** #4 AND #5

**African Index Medicus -** <https://search.bvsalud.org/aimafro/>

**#1** (mh:Nigeria OR ti:Nigeria* OR ab:Nigeria*)

**#2** (mh:"Genetic Diseases, Inborn" OR Genetic* OR Gene OR Genes OR Hereditar* OR Single-gene OR "Single gene" OR Congenital OR inherited OR "birth defect*" OR "congenital anomal*" OR "congenital malformation*" OR "fetal malformation*")

#1 AND #2

**Grey literature**

**Livivo -**  <https://www.livivo.de/>

Open search

**#1** Nigeria*

**#2** OPEN SEARCH: ("Genetic Disease*" OR "Hereditary disease*" OR "Genetic disorder*" OR "Single-gene defect*" OR "Single gene defect*" OR "Congenital disorder*" OR "inherited disease*" OR "inherited disorder*" OR "genetic syndrome*" OR "genetic defect*" OR "hereditary affliction" OR "congenital abnormalit*" OR "birth defect*" OR "congenital anomal*" OR "congenital malformation*" OR "fetal malformation*")

**#3** OPEN SEARCH: (Prevalence OR Epidemiology)

**#4** #1 AND #2 AND #3

**Google Scholar -** <https://scholar.google.com/>

Nigeria genetic disease

**Google Scholar** <https://scholar.google.com/>

allintitle: Nigeria congenital anomalies

Supplemental 1: Characteristics of included studies

| **Study ID** | **State** | **Region** | **Study design** | **Study population** | **Affected** | **Participants** | **Condition** | **Overall Prevalence**  **(%)** |
| --- | --- | --- | --- | --- | --- | --- | --- | --- |
| Abbey et al. 2017 | Rivers | South-South | Cross sectional | Livebirths | 159 | 7,670 | Major CAs | 2.1 |
| Abbey et al. 2025 | Rivers | South-South | Prospective cross sectional | Pregnant women and their fetuses | 10 | 6,078 | Aneuploides | 0.2 |
| Abhulimhen-Iyoha et al. 2013 | Edo | South-South | Cross sectional | Children | 155 | 589 | Haemoglobinopathies (AS, SS and AC) | 26.3 |
| Adeboye et al. 2017 | Niger | North-Central | Prospective Cross sectional | Children aged 1 day-16 months | 46 | 396 | CAs | 11.1 |
| Adegbehingbe et al. 2009 | Osun | South-West | Cross sectional | Secondary school students aged 9-22 years. | 81 | 4,441 | Congenital MSD | 1.8 |
| Adekoya et al. 2015 | Lagos | South-West | Retrospective cross sectional | Pediatric patients at eye clinic | 40 | 412 | CAs of the eye and adnexia | 9.7 |
| Adeoti et al. 2004 | Osun | South-West | Prospective Cross sectional | Male pupils aged 2-10 years | 40 | 1,615 | Cryptorchidism | 2.5 |
| Adeyemo et al. 1994 | Oyo | South-West | Hospital-based retrospective | Children aged hours to 13 years | 531 | 19,163 | Major CAs | 2.8 |
| Adeyokunnu 1982 | Oyo | South-West | Hospital-based retrospective | Livebirths | 29 | 25,025 | Down syndrome | 1.2 |
| Airede 1992 | Plateau | North-Central | Prospective cross sectional | Live and Stillbirths | 42 | 5,977 | Neural tube defects | 0.7 |
| Ajao 2019 | Oyo | South-West | Cross sectional retrospective | Livebirths | 67 | 1,057 | CAs | 6.3 |
| Akinbodewa et al. 2021 | Ondo | South-West | Cross sectional | Adults aged 19-44 years | 25 (21 AS, 4 AC) | 102 | Sickle cell AC and AS | 25.0 |
| Akinmoladun 2021 | Oyo | South-West | Hospital-based retrospective | Fetuses scanned for routine morphology ultrasound | 14 | 2,634 | MSD | 0.5 |
| Akinmoladun et al. 2018 | Oyo | South-West | Hospital-based prospective | Fetuses and mothers aged 15-51 years | 62 pregnant women | 989 pregnant women - fetuses | CAs (62 fetuses) 59.7% major; 40.3% minor | 6.3 |
| Akinola et al. 2008 | Lagos | South-West | Hospital-based retrospective | Pregnant women and their babies | 90 | 8,753 | CAs | 1.0 |
| Ameen et al. 2016 | Kwara | North-Central | Cross sectional | University students: 15-24 years | 372 | 440 | Haemoglobinopathies (AS, AC, SS and SC) | 48.0 |
| Animasahun et al. 2015 | Lagos | South-West | Cross sectional | Children | 983 (105 ToF) | 315, 150 | Tetralogy of Fallot | 0.05 |
| Animasahun et al. 2016 | Lagos | South-West | Prospective cross sectional | Children up to 13 years | 165 | 326,662 | Tetralogy of Fallot | 0.05 |
| Animasahun et al. 2017 | Lagos | South-West | Prospective cross sectional | Children up to 13 years | 51 | 1,693 | CHD | 3.0 |
| Anyanwu et al. 2015 | Kano | North-West | Descriptive cross sectional | Livebirths at three metropolitan hospitals | 41 | 1,456 | CAs | 2.8 |
| Anyimba et al 2025 | Enugu | South-East | Hospital-based retrospective | Patients:  abdominopelvic CT scan or CT urography | 122 | 3,507 | CAs: kidney and urinary tract | 3.5 |
| Arodiwe et al. 2019 | Enugu | South-East | Cross sectional | Children (private or insurance coverage) | 154 | 35,234 | CHD | 0.4 |
| Asuquo et al. 2016 | Not reported | South-South | Hospital-based prospective | Patients:0-9 years with idiopathic talipes equinovarus | 69 patients / 106 affected feet | Not provided | Clubfoot | 0.3 |
| Bakare et al. 2009 | Osun | South-West | Population-based Prospective | Live births in 6 local government areas of Ife and Ijesha. | 43 | 624 | CAs | 6.9 |
| Barnicot 1953 | Lagos | South-West | Cross sectional | School children | 5 | 14,292 in Lagos; 4,862 in Benin City | Albinism | 0.04 (Lagos: 1 in 2,858); 0.02 (Benin City: 1 in 4,862) |
| Bodunde et al. 2006 | Ogun | South-West | Hospital-based retrospective | Children | 105 | 14,389 | Congenital eye disease | 0.7 |
| Burnham-Marusich et al. 2016 | Enugu | South-East | Cohort | Pregnant women and male partners aged 25-35 years | 750 (SCT)  4 (SCD) | 5,545 | Sickle Cell Trait (SCT) and SCD | SCT: 13.5; SCD: 0.07 |
| Butali et al. 2014 | Kano, Borno, Yobe, Gombe, Lagos, Enugu | North-West, North-East, South-West, South-East | Cross sectional | Patients: Smile Train treatment centers | 2,197 | 4,600,000 | Orofacial cleft | 0.05 |
| Chinawa et al. 2013 | Enugu | South-East | Cross-sectional | Children from 6 months to 10 years | 16 | 31,795 | Complex CHD | 0.05 |
| Chinawa et al. 2013 | Enugu | South-East | Cross sectional | Children (aged 6 months-10 years) attending UNTH | 71 | 31,795 | CHD | 0.2 |
| Chukwubuike et al. 2020 | Enugu | South-East | Hospital-based prospective | Livebirths | 166 | 9,492 | CAs | 1.7 |
| Egesie et al. 2008 | Plateau | North-Central | Cross sectional | Healthy male donors aged 20-49 | 26 | 126 | G6PD deficiency | 20.6 |
| Egesie et al. 2013 | Plateau | North-Central | Cross sectional | Adults 20-49 years | 27 | 130 | Sickle Cell Trait and G6PD Deficiency | 20.8 |
| Ekanem et al. 2008 | Cross River and Akwa Ibom | South-South | Cross sectional | Infants | 452 | 127,929 | CAs | 0.4 |
| Eke et al. 2016 | Enugu | South-East | Cross sectional | Infants | 72 | 7,329 | Central Nervous System CAs | 1.0 |
| Ekure et al. 2020 | Lagos | South-West | Community-based cross sectional | Primary and secondary school students | 27 | 4,107 | CHD | 0.7 |
| Ekwochi et al. 2018 | Enugu | South-East | Hospital-based prospective | Livebirths | 38 | 5,830 | CAs | 0.7 |
| Ekwunife et al. 2017 | Anambra | South-East | Prospective cross sectional | Infants that were treated at the hospital | 108 | 5,010 | CAs | 2.2 |
| Emeka and Ikemefuna 2021 | Enugu | South-East | Observational | Livebirths | 93 | 17,241 | Genitourinary CAs | 0.5 |
| Engwa et al. 2017 | Enugu | South-East | Cross sectional | Pregnant women aged 23-35 years | 57 | 95 | G6PD deficiency | 60.0 |
| Eseigbe et al. 2018 | Benue | North-Central | Hospital-based retrospective | Neonates admitted at the hospital | 73 | 785 | Congenital anomalies of CNS | 9.3 |
| Ezenwosu et al. 2021 | Benue | North-Central | Cross sectional | Pregnant women + partners (aged 13-59 years); HBI 12 LGAs | 1909 (SCT)  6 (SCD) | 10,167 | Sickle cell disease and trait | SCT: 1.1; SCD: 0.06 |
| Fakorede et al. 2022 | Lagos | South-West | Cross sectional | University students from the three major Nigerian ethnic groups aged 18-39 | 34 | 1,191 | Colour vision deficiency | 2.9 |
| Fleming et al. 1979 | Kano | North-West | Cohort | Adults and children | 817 | 2,742 | Haemoglobinopathies | 29.8 (0.2 SCD alone) |
| Gilles et al. 1967 | Oyo | South-West | Case control | Children aged 6 months to 4 years with suspected falciparum malaria | 100 | 300 (Severe malaria: 100; Control: 200) | G6PD Deficiency and Sickle cell trait | G6PD Deficiency (13.3), HbAC (6.7), HbAS (13.3) |
| Ige et al. 2021 | Plateau | North-Central | Cross sectional | Neonates aged one week or younger | 111 | 3,857 | CHD | 2.9 |
| Ikemefuna et al. 2021 | Enugu | South-East | Hospital-based observational | Livebirths | 79 | 15,820 | Congenital anomalies of CNS | 0.5 |
| Iregbulem 1982 | Enugu | South-East | Hospital-based prospective | Infants | 8 | 21,624 | Cleft lip and palate | 0.04 |
| Isaac et al. 2013 | Sokoto | North-West | Hospital-based prospective | Children | 17 | 118 | G6PD deficiency | 11.4 |
| John-Olabode et al. 2021 | Lagos | South-West | Cross sectional | Pregnant women 15-49 years antenatal care in two hospitals. | 3 | FVL: 397; r F2 G20210A: 389 | Inherited thrombophilias | 0.8 (FVL) |
| Lawan 2008 | Kano | North-West | Hospital-based retrospective | Children less than 10 years old | 69 (109 eyes) | 4,431 | Congenital eye and adnexial anomalies | 1.6 |
| Niandat and Okoli 2025 | Plateau | North-Central | Hospital-based cross sectional | Children aged 0-5 years | 40 | 100 | G6PD deficiency | 40.0 |
| Nnaji et al. 2013 | Anambra | South-East | Cross sectional | Prospective couples aged 23-75 | 116 | 424 | SCD and Traits | 27.4 (0.9 SCD) |
| Nwabuko 2015 | Rivers | South-South | Hospital-based retrospective | Pregnant women | 7,161 | 35,976 | SCD | 19.9 |
| Nwabuko et al. 2022 | Abia | South-East | Cross sectional | Patients: children and adults aged 7 months-49 years | 138 | 8,457 | SCD | 1.6; 1% Pediatric 0.6% Adult |
| Obu et al. 2012 | Enugu | South-East | Cross sectional | Livebirths | 17 | 607 | CAs | 2.8 |
| Odunvbun et al. 2008 | Edo | South-South | Cross sectional | Newborns | 139 | 644 | HbSS and HbSC | 3.0 |
| Okeke and Osegbe 2001 | Anambra | South-East | Prospective community-based cross sectional | Males aged 5-13 years: primary schools | 9 | 1,096 | Cryptorchidism | 0.8 |
| Okonkwo et al. 2011 | Edo | South-South | Hospital based prospective | Livebirths  Special Care Unit University of Benin Teaching Hospital | 85 | 1,513 | CAs | 5.6 |
| Okunola et al. 2020 | Ekiti | South-West | Hospital-based retrospective | All deliveries | 5 | 3,789 | Anencephaly | 0.1 |
| Oloyede et al. 2020 | Ogun | South-West | Retrospective cross sectional | Pregnant women and their fetuses | 78 | 9,698 | CAs | 0.8 |
| Olusanya, 2012 | Lagos | South-West | Cohort | Infants | 340  74 | 3,196 | Microcephaly macrocephaly | 10.6  2.3 |
| Oluwafemi et al. 2019 | Ondo | South-West | Cross sectional | Infants | 39 | 8,307 | Major CAs | 0.5 |
| Oluwole et al. 2020 | Lagos | South-West | Cross sectional | Mothers aged 18 and above, and their infants aged between 2-10 weeks. | 65 (3 SCD) | 250 | SCD and trait | 26 (1.2 SCD) |
| Otaigbe 2014 | Rivers | South-South | Cross-sectional | Children | 332 | 23,124 | CHD | 1.4 |
| Oyinbo et al. 2009 | Bayelsa | South-South | Cross sectional | Residents: Bayelsa state. | 262 | 2,000 | CAs | 13.1 |
| Ozoemena and Mbah 2006 | Enugu | South-East | Cross-sectional | Male students at least 10 years old at secondary schools; urban rural | 416 | 6,226 | CAs male external genitalia | 6.7 |
| Sadoh et al. 2021 | Edo | South-South | Descriptive cross sectional | Liveborn delivered in the hospital at postnatal or neonatal wards | 41 | 2,849 | CHD | 1.4 |
| Saganuwan 2016 | Sokoto | North-West | Cross sectional | SCD Patients: haematological unit of the hospital | 197 | 319 | Haemoglobinopathies (AS, SS, SC, SS+F) | 61.8 |
| Shonde-Adebola et al. 2018 | Oyo | South-West | Cross sectional | Healthy youths aged 15-35 years | 4 | 182 | Von Willebrand Disease | 2.2 |
| Stephen et al. 2018 | Plateau | North-Central | Hospital-based retrospective | Infants, children and adolescents (0-18 years) | 82 | 3,047 | SCD | 2.7 |
| Sunday-Adeoye 2007 | Ebonyi | South-East | Cross sectional | Singletons or twins’ live births | 315 singletons / 58 twins | 32,206 singletons / 1,453 twins | CAs | 1.1 |
| Ugwu et al. 2008 | Plateau | North-Central | Hospital-based retrospective | Infants | 37 | 2,891 | Neural tube defect | 1.3 |
| Ujuanbi et al. 2016 | Rivers | South-South | Cross sectional | School children aged 5-14 years | 31 | 1,712 | CHD | 1.8 |
| Umoh et al. 2010 | Akwa Ibom | South-South | Cross sectional | Individuals who underwent haemoglobin genotype testing | 125 | 8,097 | HbSS and HbSC | 1.5 |
| Wagula and Udo-Peretomode 2020 | Rivers | South-South | Retrospective cross sectional | Livebirths | 837 | 21,738 | CAs | 3.9 |
| Williams et al. 2013 | Oyo | South-West | Cross sectional | Children aged 1 month to 15 years | 172 | 1,134 (Analysed 1,122) | G6PD deficiency | 15.3 |
| Yilgwan et al. 2012 | Plateau | North-Central | Community-based cross sectional | Children aged 6-12 years | 3 | 418 | CHD | 0.7 |

Legend CAs: Congenital Anomalies; CHD = Congenital Heart Defects; SCD = Sickle Cell Disease; SCT = Sickle Cell Trait; FVL G1691A = Factor V Leiden mutation; F2 G20210A = Prothrombin gene mutation; MSD = Musculoskeletal defects; CNS = Central Nervous System

Supplemental 2: Congenital anomalies based on anatomical sites, studies, regions, and prevalence

| **Anatomical site** | **Study ID** | **State (Region)** | **Prevalence (%)** |
| --- | --- | --- | --- |
| Central Nervous System (Head) | Adeboye et al. 2016 | Niger (North-Central) | 2.0 |
|  | Airede 1992 | Plateau (North-Central) | 0.7 |
|  | Eseigbe et al. 2018 | Benue (North-Central) | 3.6 |
|  | Anyanwu et al. 2015 | Kano (North-West) | 0.7 |
|  | Chukwubuike et al. 2020 | Enugu (South-East) | 0.6 |
|  | Eke et al. 2016 | Enugu (South-East) | 1.0 |
|  | Ekwochi et al. 2018 | Enugu (South-East) | 0.2 |
|  | Ekwunife et al. 2017 | Anambra (South-East) | 0.4 |
|  | Ikemefuna et al. 2021 | Enugu (South-East) | 0.5 |
|  | Obu et al. 2012 | Enugu (South-East) | 1.8 |
|  | Sunday-Adeoye et al. 2007 | Ebonyi (South-East) | 0.2 |
|  | Abbey et al. 2017 | Rivers (South-South) | 0.6 |
|  | Ekanem et al. 2008 | Cross River and Akwa Ibom (South-South) | 0.09 |
|  | Okonkwo et al. 2011 | Edo (South-South) | 1.6 |
|  | Ugwu et al. 2008 | Rivers (South-South) | 1.3 |
|  | Wagwula and Udo-Peretomode 2020 | Rivers (South-South) | 0.8 |
|  | Adeyemo et al. 1994 | Oyo (South-West) | 0.7 |
|  | Ajao and Adeoye 2019 | Oyo (South-West) | 0.4 |
|  | Akinmoladun et al. 2018 | Oyo (South-West) | 0.9 |
|  | Akinola et al. 2008 | Lagos (South-West) | 0.5 |
|  | Bakare et al. 2009 | Osun (South-West) | 1.3 |
|  | Okunola et al. 2020 | Ekiti (South-West) | 0.1 (Anencephaly) |
|  | Oloyede et al. 2020 | Ogun (South-West) | 0.4 |
|  | Olusanya 2012 | Lagos (South-West) | 13.0 |
|  | Oluwafemi et al. 2019 | Ondo (South-West) | 0.2 |
|  |  |  |  |
| Chest | Bakare et al. 2009 | Osun (South-West) | 0.2 |
|  |  |  |  |
| Cardiovascular | Adeboye et al. 2016 | Niger (North-Central) | 0.8 |
|  | Eseigbe et al. 2018 | Benue (North-Central) | 0.5 |
|  | Ige et al. 2021 | Plateau (North-Central) | 2.9 |
|  | Yilgwan et al. 2012 | Plateau (North-Central) | 0.7 |
|  | Arodiwe et al. 2019 | Enugu (South-East) | 0.4 |
|  | Chinawa et al. 2013 | Enugu (South-East) | 0.05 (two or more heart anomalies) |
|  | Chinawa et al. 2013 | Enugu (South-East) | 0.2 |
|  | Ekwochi et al. 2018 | Enugu (South-East) | 0.2 |
|  | Abbey et al. 2017 | Rivers (South-South) | 0.2 |
|  | Ekanem et al. 2008 | Cross River and Akwa Ibom (South-South) | 0.001 |
|  | Okonkwo et al. 2011 | Edo (South-South) | 0.2 |
|  | Otaigbe et al. 2014 | Rivers (South-South) | 1.4 |
|  | Sadoh et al. 2021 | Edo (South-South) | 1.4 |
|  | Ujuanbi et al. 2016 | Rivers (South-South) | 1.8 |
|  | Wagwula and Udo-Peretomode 2020 | Rivers (South-South) | 1.1 |
|  | Adeyemo et al. 1994 | Oyo (South-West) | 0.8 |
|  | Ajao and Adeoye 2019 | Oyo (South-West) | 1.0 |
|  | Akinmoladun et al. 2018 | Oyo (South-West) | 0.5 |
|  | Animasahun et al. 2015 | Lagos (South-West) | 0.05 (TOF) |
|  | Animasahun et al. 2016 | Lagos (South-West) | 0.05 (TOF) |
|  | Animasahun et al. 2017 | Lagos (South-West) | 3.0 |
|  | Akinola et al. 2008 | Lagos (South-West) | 0.07 |
|  | Ekure et al. 2020 | Lagos (South-West) | 0.7 |
|  | Oloyede et al. 2020 | Ogun (South-West) | 0.02 |
|  |  |  |  |
| Gastrointestinal | Adeboye et al. 2016 | Niger (North-Central) | 5.8 |
|  | Eseigbe et al. 2018 | Benue (North-Central) | 3.2 |
|  | Anyanwu et al. 2015 | Kano (North-West) | 0.3 |
|  | Chukwubuike et al. 2020 | Enugu (South-East) | 0.1 |
|  | Ekwochi et al. 2018 | Enugu (South-East) | 0.4 |
|  | Ekwunife et al. 2017 | Anambra (South-East) | 0.6 |
|  | Obu et al. 2012 | Enugu (South-East) | 0.3 |
|  | Obu et al. 2012 | Enugu (South-East) | 0.2 (Anorectal) |
|  | Sunday-Adeoye et al. 2007 | Ebonyi (South-East) | 0.1 |
|  | Abbey et al. 2017 | Rivers (South-South) | 0.4 |
|  | Ekanem et al. 2008 | Cross River and Akwa Ibom (South-South) | 0.02 |
|  | Okonkwo et al. 2011 | Edo (South-South) | 2.0 |
|  | Wagwula and Udo-Peretomode 2020 | Rivers (South-South) | 0.3 |
|  | Adeyemo et al. 1994 | Oyo (South-West) | 0.5 |
|  | Ajao and Adeoye 2019 | Oyo (South-West) | 1.5 |
|  | Akinmoladun et al. 2018 | Oyo (South-West) | 0.6 |
|  | Akinola et al. 2008 | Lagos (South-West) | 0.1 |
|  | Bakare et al. 2009 | Osun (South-West) | 1.1 |
|  | Oloyede et al. 2020 | Ogun (South-West) | 0.2 |
|  | Oluwafemi et al. 2019 | Ondo (South-West) | 0.1 |
|  |  |  |  |
| Musculoskeletal | Adeboye et al. 2016 | Niger (North-Central) | 2.0 |
|  | Eseigbe et al. 2018 | Benue (North-Central) | 2.3 |
|  | Anyanwu et al. 2015 | Kano (North-West) | 0.2 |
|  | Chukwubuike et al. 2020 | Enugu (South-East) | 0.8 |
|  | Ekwochi et al. 2018 | Enugu (South-East) | 1.1 |
|  | Ekwunife et al. 2017 | Anambra (South-East) | 0.3 |
|  | Sunday-Adeoye et al. 2007 | Ebonyi (South-East) | 0.6 |
|  | Abbey et al. 2017 | Rivers (South-South) | 0.1 |
|  | Asuquo et al. 2016 | South-South | 0.3 (Club foot) |
|  | Ekanem et al. 2008 | Cross River and Akwa Ibom (South-South) | 0.1 |
|  | Okonkwo et al. 2011 | Edo (South-South) | 0.8 |
|  | Oyinbo et al. 2009 | Bayelsa (South-South) | 8.8 |
|  | Wagwula and Udo-Peretomode 2020 | Rivers (South-South) | 1.0 |
|  | Adegbehingbe et al. 2009 | Osun (South-West) | 1.8 |
|  | Ajao and Adeoye 2019 | Oyo (South-West) | 0.8 |
|  | Akinmoladun et al. 2018 | Oyo (South-West) | 0.2 |
|  | Akinmoladun 2021 | Oyo (South-West) | 0.5 |
|  | Akinola et al. 2008 | Lagos (South-West) | 0.1 |
|  | Bakare et al. 2009 | Osun (South-West) | 3.4 |
|  | Oloyede et al. 2020 | Ogun (South-West) | 0.1 |
|  | Oluwafemi et al. 2019 | Ondo (South-West) | 0.1 |
|  |  |  |  |
| Genitourinary | Adeboye et al. 2016 | Niger (North-Central) | 1.3 |
|  | Eseigbe et al. 2018 | Benue (North-Central) | 1.1 |
|  | Anyanwu et al. 2015 | Kano (North-West) | 0.7 |
|  | Anyimba et al. 2025 | Enugu (South-East) | 3.5 |
|  | Chukwubuike et al. 2020 | Enugu (South-East) | 0.2 |
|  | Ekwochi et al. 2018 | Enugu (South-East) | 0.2 |
|  | Ekwunife et al. 2017 | Anambra (South-East) | 0.5 |
|  | Emeka and Ikemefuna 2021 | Enugu (South-East) | 0.5 |
|  | Okeke and Osegbe 2001 | Anambra (South-East) | 0.8 |
|  | Ozoemena and Mbah 2006 | Enugu (South-East) | 6.7 |
|  | Sunday-Adeoye et al. 2007 | Ebonyi (South-East) | 0.05 |
|  | Abbey et al. 2017 | Rivers (South-South) | 0.2 |
|  | Ekanem et al. 2008 | Cross River and Akwa Ibom (South-South) | 0.07 |
|  | Okonkwo et al. 2011 | Edo (South-South) | 0.6 |
|  | Wagwula and Udo-Peretomode 2020 | Rivers (South-South) | 0.04 |
|  | Adeyemo et al. 1994 | Oyo (South-West) | 0.3 |
|  | Ajao and Adeoye 2019 | Oyo (South-West) | 0.8 |
|  | Adeoti et al. 2004 | Osun (South-West) | 2.5 (Cryptorchidism) |
|  | Akinmoladun et al. 2018 | Oyo (South-West) | 0.6 |
|  | Akinola et al. 2008 | Lagos (South-West) | 0.09 |
|  | Bakare et al. 2009 | Osun (South-West) | 1.4 |
|  | Oloyede et al. 2020 | Ogun (South-West) | 0.1 |
|  |  |  |  |
| Craniofacial | Anyanwu et al. 2015 | Kano (North-West) | 0.1 |
|  | Chukwubuike et al. 2020 | Enugu (South-East) | 0.04 |
|  | Ekwunife et al. 2017 | Anambra (South-East) | 0.4 |
|  | Iregbulem 1982 | Enugu (South-East) | 0.04 |
|  | Obu et al. 2012 | Enugu (South-East) | 0.3 |
|  | Sunday-Adeoye et al. 2007 | Ebonyi (South-East) | 0.08 |
|  | Abbey et al. 2017 | Rivers (South-South) | 0.09 |
|  | Ekanem et al. 2008 | Cross River and Akwa Ibom (South-South) | 0.05 |
|  | Oyinbo et al. 2009 | Bayelsa (South-South) | 1.3 |
|  | Wagwula and Udo-Peretomode 2020 | Rivers (South-South) | 0.5 |
|  | Adeyemo et al. 1994 | Oyo (South-West) | 0.2 |
|  | Ajao and Adeoye 2019 | Oyo (South-West) | 0.2 |
|  | Akinmoladun et al. 2018 | Oyo (South-West) | 0.1 |
|  | Akinola et al. 2008 | Lagos (South-West) | 0.06 |
|  | Oluwafemi et al. 2019 | Ondo (South-West) | 0.02 |
|  | Butali et al. 2014 | Multiple sites | 0.05 |
|  |  |  |  |
| Ocular | Lawan 2008 | Kano (North-West) | 1.6 |
|  | Sunday-Adeoye et al. 2007 | Ebonyi (South-East) | 0.03 |
|  | Ekanem et al. 2008 | Cross River and Akwa Ibom (South-South) | 0.01 |
|  | Adekoya et al. 2015 | Lagos (South-West) | 9.7 |
|  | Ajao and Adeoye 2019 | Oyo (South-West) | 0.2 |
|  | Bodunde et al. 2006 | Ogun (South-West) | 0.7 |
|  |  |  |  |
| Skin/Integumentary | Adeboye et al. 2016 | Niger (North-Central) | 1.5 (Head & Neck) |
|  | Anyanwu et al. 2015 | Kano (North-West) | 0.4 |
|  | Ekwochi et al. 2018 | Enugu (South-East) | 0.2 |
|  | Abbey et al. 2017 | Rivers (South-South) | 0.03 |
|  | Bakare et al. 2009 | Osun (South-West) | 0.2 |
|  |  |  |  |
| Pulmonary/Respiratory | Ekanem et al. 2008 | Cross River and Akwa Ibom (South-South) | 0.02 |
|  | Ajao and Adeoye 2019 | Oyo (South-West) | 0.1 |
|  | Akinola et al. 2008 | Lagos (South-West) | 0.03 |
|  | Oloyede et al. 2020 | Ogun (South-West) | 0.02 |
|  |  |  |  |
| Chromosomal | Adeboye et al. 2016 | Niger (North-Central) | 0.3 (Down) |
|  | Anyanwu et al. 2015 | Kano (North-West) | 0.07 (Down) |
|  | Ekwunife et al. 2017 | Anambra (South-East) | 0.06 (Down) |
|  | Obu et al. 2012 | Enugu (South-East) | 0.2 (Patau) |
|  | Abbey et al. 2017 | Rivers (South-South) | 0.07 (Edwards) |
|  | Abbey et al. 2017 | Rivers (South-South) | 0.05 (Down) |
|  | Abbey et al. 2025 | Rivers (South-South) | 0.1 (Down) |
|  | Abbey et al. 2025 | Rivers (South-South) | 0.05 (Edwards) |
|  | Ekanem et al. 2008 | Cross River and Akwa Ibom (South-South) | 0.01 (Down) |
|  | Okonkwo et al. 2011 | Edo (South-South) | 0.3 (Down) |
|  | Okonkwo et al. 2011 | Edo (South-South) | 0.07 (Edwards) |
|  | Wagwula and Udo-Peretomode 2020 | Rivers (South-South) | 0.08 (Down) |
|  | Adeyemo et al. 1994 | Oyo (South-West) | 0.07 (Down) |
|  | Adeyokunnu 1982 | Oyo (South-West) | 0.1 (Down) |
|  | Ajao and Adeoye 2019 | Oyo (South-West) | 0.5 (Down) |
|  | Akinola et al. 2008 | Lagos (South-West) | 0.01 (Down) |
|  | Animasahun et al. 2015 | Lagos (South-West) | 0.002 (Down) |
|  | Animasahun et al. 2015 | Lagos (South-West) | 0.001 (Turner) |
|  | Oluwafemi et al. 2019 | Ondo (South-West) | 0.01 (Turner) |
|  |  |  |  |
| Syndrome | Anyanwu et al. 2015 | Kano (North-West) | 0.07 (Beckwith-Wiedemann) |
|  | Abbey et al. 2017 | Rivers (South-South) | 0.01 (Beckwith-Wiedemann) |
|  | Abbey et al. 2017 | Rivers (South-South) | 0.01 (Prune Belly) |
|  | Oyinbo et al. 2009 | Bayelsa (South-South) | 3.0 (Amniotic Band sequence) |
|  | Animasahun et al. 2015 | Lagos (South-West) | 0.0003 (CATCH 22) |
|  | Oluwafemi et al. 2019 | Ondo (South-West) | 0.01 (Noonan) |
|  | Oluwafemi et al. 2019 | Ondo (South-West) | 0.01 (Pierre Robin) |
|  | Butali et al. 2014 | Multiple sites | 0.2 (Apert) |
|  | Butali et al. 2014 | Multiple sites | 0.2 (Van der Woude) |
|  | Oluwafemi et al. 2019 | Ondo (South-West) | 0.04 (Unclassified) |
|  |  |  |  |
| Fetal tumour  (Sacrococcygeal teratoma and nephroblastoma) | Ekwunife et al. 2017 | Anambra (South-East) | 0.06 |
|  | Abbey et al. 2017 | Rivers (South-South) | 0.03 |
|  |  |  |  |
| Others | Abbey et al. 2017 | Rivers (South-South) | 0.01 (Hypothyroidism) |
|  | Bakare et al. 2009 | Osun (South-West) | 0.2 |
|  |  |  |  |
| Multiple | Abbey et al. 2017 | Rivers (South-South) | 0.3 |
|  | Adeyemo et al. 1994 | Oyo (South-West) | 0.08 |
|  | Ajao and Adeoye 2019 | Oyo (South-West) | 0.5 |
|  | Akinmoladun et al. 2018 | Oyo (South-West) | 0.8 |
| Undetermined/Multiple | Anyanwu et al. 2015 | Kano (North-West) | 0.2 |
